# A Monodisperse Population Balance Model for Nanoparticle Agglomeration in the Transition Regime

**DOI:** 10.3390/ma14143882

**Published:** 2021-07-12

**Authors:** Georgios A. Kelesidis, M. Reza Kholghy

**Affiliations:** 1 Department of Mechanical and Process Engineering, Eidgenössische Technische Hochschule Zürich, Sonneggstrasse 3, 8092 Zürich, Switzerland; gkelesidis@ptl.mavt.ethz.ch; 2 Department of Mechanical and Aerospace Engineering, Carleton University, 1125 Colonel by Drive, Ottawa, ON K1S 5B6, Canada

**Keywords:** agglomeration, transition regime, population balance model, discrete element model, fractal-like structure

## Abstract

Nanoparticle agglomeration in the transition regime (e.g. at high pressures or low temperatures) is commonly simulated by population balance models for volume-equivalent spheres or agglomerates with a constant fractal-like structure. However, neglecting the fractal-like morphology of agglomerates or their evolving structure during coagulation results in an underestimation or overestimation of the mean mobility diameter, $d_{m}$, by up to 93 or 49%, repectively. Here, a monodisperse population balance model (MPBM) is interfaced with robust relations derived by mesoscale discrete element modeling (DEM) that account for the realistic agglomerate structure and size distribution during coagulation in the transition regime. For example, the DEM-derived collision frequency, β, for polydisperse agglomerates is 82 ± 35% larger than that of monodisperse ones and in excellent agreement with measurements of flame-made TiO_2_ nanoparticles. Therefore, the number density, $N_{Ag}$, mean, $d_{m},$ and volume-equivalent diameter, $d_{v}$, estimated here by coupling the MPBM with this β and power laws for the evolving agglomerate morphology are on par with those obtained by DEM during the coagulation of monodisperse and polydisperse primary particles at pressures between 1 and 5 bar. Most importantly, the MPBM-derived $N_{Ag}$, $d_{m}$, and $d_{v}$ are in excellent agreement with the data for soot coagulation during low temperature sampling. As a result, the computationally affordable MPBM derived here accounting for the realistic nanoparticle agglomerate structure can be readily interfaced with computational fluid dynamics in order to accurately simulate nanoparticle agglomeration at high pressures or low temperatures that are present in engines or during sampling and atmospheric aging.

## 1. Introduction

Nanoparticles made by gas-phase manufacturing or emitted by incomplete combustion of fossil fuels are abundant in our everyday lives [1]. Incipient nanoparticles grow by coagulation, sintering and surface growth. Inception [2], surface growth [3] and sintering [4] are active only in a narrow window of time, when the temperature is very high. Thus, coagulation is the dominant process in controlling nanoparticle morphology and number concentration, forming fractal-like agglomerates [5]. Such agglomerates are often present at very high concentrations during the gas-phase synthesis of carbon black, ceramic (TiO_2_ and SiO_2_) and metallic (Ni, Fe and Cu) powders, as well as soot emissions from engines, fires and volcanic plumes [1]. At high pressures or low temperatures that are present in engines [6] or during sampling [7] and atmospheric aging of aerosols [8], these agglomerates are formed by coagulation in the transition regime.

Agglomeration dynamics could be simulated by population balance models if an accurate collision frequency, β, and realistic particle morphologies are employed [5]. For example, sectional population balance models (SPBMs) are used to simulate agglomeration in the transition regime, with β obtained from the harmonic mean of those in the free molecular and continuum regimes [9] based on the agglomerate gyration, $d_{g}$, mobility, $d_{m}$, and primary particle, $d_{p}$, diameters. The agglomerate structure, quantified by the relation between $d_{g}$ and $d_{m}$ [10], changes depending on the number [11], diameter and polydispersity of their constituent primary particles [12]. Accounting for the evolving agglomerate structure with SPBM is not trivial, as multiple equations per section need to be solved [13,14]. Therefore, models for the combustion synthesis of nanomaterials assume that $d_{m}=d_{g}=d_{p}\;n_{p}^{0.56}$, based on a constant fractal dimension, $D_{f}=1.8$ [15,16] often with a constant value for $d_{p}$ [17,18]. Similarly, climate models simulate the coagulation dynamics of aerosols assuming that the spheres have the same volume-equivalent diameter, *d_v_*, with nanoparticle agglomerates [19].

With mesoscale discrete element modeling (DEM), the evolution of particle size distribution [3], morphology [10] and collision frequency [20] can be obtained from first principles. For example, it was recently shown that DEM-derived agglomerates reach a quasi-self-preserving size distribution (SPSD) with a mobility-based geometric standard deviation, *σ_g,m_* = 1.48 ± 0.03 [21], that is narrower than the SPSD attained in the free molecular regime [12] and in excellent agreement with data for SiO_2_[22] and soot [23,24] agglomerates. The DEM-derived collision frequency of polydisperse agglomerates at their quasi-SPSD was on average 82% higher than that of monodisperse ones, regardless of the chemical bonding and polydispersity of their constituent primary particles [21]. This DEM-derived collision frequency enhancement is on par with those measured from flame-made TiO_2_ nanoparticle agglomerates in the transition regime [21]. Furthermore, power laws relating the mobility diameter normalized by the primary particle diamater, $d_{m}/d_{p}$, or gyration diameter, $d_{m}/d_{g}$, with the number of primary particles per agglomerate, $n_{p}$, are derived by DEM simulations in the free molecular and transition regimes [3]. Such power laws can be used in population balance models to estimate accurately the evolving structure of nanoparticles during agglomeration in the transition regime.

Sectional models have been interfaced with computational fluid dynamics (CFD) in order to explain soot formation in diffusion flames [13,14] or predict agglomeration of soot nanoparticles from diesel engine exhausts [7,25]. Finite element methods have also been coupled with SPBMs to simulate coagulation of spheres [26], fractal-like agglomerates [27] and linear stacks (rouleaux) [28]. However, both SPBM and mesoscale simulations are computationally expensive and interfacing them with CFD is not trivial. Monodisperse population balance models (MPBMs) are computationally affordable and easy to use [16,29]. However, they apply best when particles have attained their SPSD and asymptotic fractal-like structure by coagulation [30]. This is typically the case when high concentrations of nanoparticles coagulate and rapidly attain their quasi-SPSD [21]. Kruis et al. [16] used a three equation MPBM assuming monodisperse agglomerate and primary particle size distributions to track their coagulation and sintering and compared it to a two dimensional SPBM [31]. The influence of the fractal-like agglomerate structure on its β was accounted for by estimating the agglomerate collision diameter, $d_{c}$, using $D_{f}$, $d_{p}$ and $n_{p}$. The MPBM predictions agreed well with those of the SPBM for the equivalent primary particle diameter. However, the agglomerate concentration was overpredicted, because the enhancement of β due to the polydispersity of the agglomerate size distribution was not considered. Goudeli et al. [11] interfaced a MPBM with an evolving DEM-derived $D_{f}$ to elucidate the agglomerate dynamics during coagulation and sintering in the free molecular regime. Neglecting the evolution of $D_{f}$ hardly affected $d_{p}$, but overpredicted the agglomerate collision diameter up to 30% during the transition from hard- to soft-agglomeration (e.g., when the characteristic collision and coalescence times were comparable) [11]. In this regard, the MPBM for coagulation and surface growth [32] was coupled with DEM-derived power laws and coagulation rates in order to accurately describe the dynamics of soot nanoparticles in the free molecular regime. Extending this MPBM for coagulation in the transition regime is essential for the design of cleaner combustion engines, robust sampling devices for flame-made nanomaterials and accurate climate models for the estimation of the aerosol environmental impact.

The objective of this work is to develop an accurate but computationally affordable MPBM for nanoparticle agglomeration in the transition regime accounting for the evolving agglomerate structure and size distribution. Thus, the DEM-derived quasi-self-preserving size distributions of agglomerates [21] and data-proven power laws governing their structure during their formation in the transition regime [10] are interfaced with a simple and accurate MPBM of their coagulation dynamics. In particular, the coagulation of nanoparticles is elucidated by a MPBM at low and high temperatures and pressures that are relevant during particle formation in engines [6] or sampling [7] and atmospheric aging of aerosols [8]. The collision frequency, number concentration, mobility and volume-equivalent diameters are validated with DEM simulations and data of soot nanoparticles at identical conditions.

## 2. Theory

### 2.1. Discrete Element Modeling and Agglomerate Structure 

Ballistic and Brownian coagulation dynamics of nanoparticles in the absence of rotation, convection, deposition, van der Waals, electric or hydrodynamic forces are simulated by DEM of agglomeration in the free molecular and transition regimes [20]. This is a serial, event-driven algorithm implemented in C++, as detailed in [20]. In brief, spheres with mean diameter, $d_{p}$, of 20 nm and volume fraction of 1 ppm are randomly distributed in a cubic cell at 1 bar and 1830 K. The bulk primary particle density is set to $1800\;\mathrm{kg}/m^{3}$, which is commonly used to simulate the dynamics of mature soot [3] and is close to that of SiO_2_ [11]. Particles are in equilibrium with the surrounding gas and change direction after each collision or after traveling their persistence length [33]. The time between collisions is calculated with an event-driven method [34]. Particles stick to each other after collisions and the trajectory of the newly formed agglomerate is calculated by the momentum conservation principle. The surrounding gas pressure, *P*, is increased from 1 to 5 bar to simulate conditions close to those in combustion engines [6]. The geometric standard deviation, $\sigma_{g,p}$, of the initial particle size distribution is also varied from 1 to 1.5 in order to be consistent with the measured soot [35] and metal oxide [22] primary particle size distributions.

The mobility diameter, $d_{m}$, of DEM-derived agglomerates in the free molecular and transition regimes is related to their number of primary particles per agglomerate, $n_{p}$, and $d_{p}$ by [10]:

(1) $$\frac{d_{m}}{d_{p}}=n_{p}{}^{0.45}$$

and to their diameter of gyration, $d_{g}$, by [10]:

(2) $$\frac{d_{m}}{d_{g}}=\{\begin{array}{c} n_{p}^{-0.2}+0.4\;\;\;\;\;\;\;\;,\;n_{p}>1.8\\ \sqrt{5/3}\;\;\;\;\;\;\;\;\;\;\;\;\;\;\;\;\;\;\;\;\;,\;n_{p}\leq 1.8 \end{array}$$

These power laws are obtained for soot particles formed in flames with maximum soot volume fractions spanning two orders of magnitude [10]. Furthermore, Equation (1) has been validated with data of soot, SiO_2_, ZrO_2_, Au and Ag aerosols [36].

**Figure 1:**
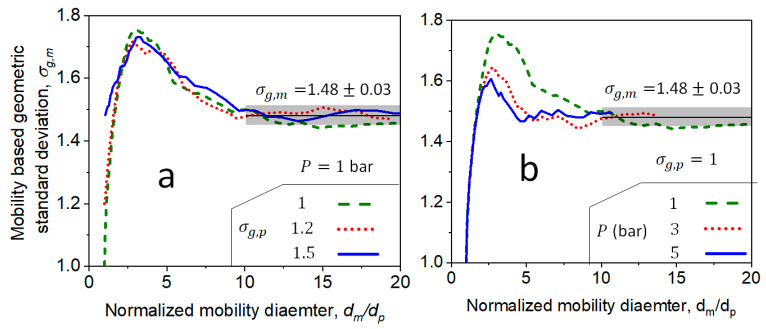
Evolution of geometric standard deviation of agglomerate mobility diameter, $\sigma_{g,m},$ as a function of the normalized mobility diameter, $d_{m}/d_{p}$, during coagulation (**a**) at P=1 bar for agglomerates consisting of polydisperse primary particles with $1\leq \sigma_{g,p}\leq 1.5$, and (**b**) for agglomerates of monodisperse primary particles coagulating at different pressures, 1≤P≤5 bar.

The evolution of the detailed DEM-derived *d_m_* distribution from the free molecular to the transition regime has been validated with data of organic and inorganic nanoparticles from premixed [3], diffusion [21] and spray flames [21]. Figure 1 shows the DEM-derived geometric standard deviation, $\sigma_{g,m}$, of the *d_m_* distribution as a function of the normalized mean, *d_m_*/*d_p_,* of agglomerates, with primary particles having $\sigma_{g,p}$ = 1 (a, b: broken lines), 1.2 (a: dotted line) and 1.5 (a: solid line) coagulating at 1830 K, and *P* = 1 (a, b: broken lines), 3 (b: dotted line) and 5 bar (b: solid line). Small $\sigma_{g,p}$ = 1 and 1.2 are representative for organic nanoparticles (e.g., soot [3,35]), while $\sigma_{g,p}$ = 1.5 is common for inorganic nanoparticles (e.g., SiO_2_ [22] and ZrO_2_ [37]) that attain their self-preserving size distribution by coalescence. Regardless of $\sigma_{g,p}$, the $\sigma_{g,m}$ of the agglomerate size distribution increases up to 1.7 ± 0.05 in the free molecular regime at $d_{m}/d_{p}=3$, consistent with DEM simulations of agglomeration and surface growth [10]. At $d_{m}/d_{p}>3$, agglomerates coagulate in the transition regime and their $\sigma_{g,m}$ decreases, asymptotically attaining its quasi-self-preserving $\sigma_{g,m}$ = 1.48 ± 0.03 [21] (thin solid line and shaded area). Increasing *P* from 1 to 5 bar accelerates the attainment of the $\sigma_{g,m}$ = 1.48 ± 0.03, as agglomerates coagulate mostly in the transition regime at these conditions. The large $\sigma_{g,m}$ attained by coagulation in the free molecular and transition regimes enhances the agglomerate coagulation frequency, as elaborated in the next section.

### 2.2. Agglomerate Dynamics With a Monodisperse Population Balance Model

Here, a monodisperse population balance model [16] is implemented in MATLAB (R2020a, MathWorks, Inc., Natick, MA, USA) and used to describe the coagulation dynamics of agglomerates in the transition regime. As the total mass and surface area of the agglomerates are conserved, one equation is enough to track the agglomerate number density and structure. At isothermal conditions, the rate of change in the total agglomerate number concentration, $N_{Ag}$, is [5]:

(3) $$\frac{dN_{Ag}}{dt}=-\frac{1}{2}\beta_{m}N_{Ag}^{2}$$

where $\beta_{m}$ is the collision frequency of monodisperse agglomerates given by the harmonic mean [9]:

(4) $$\beta_{m}=\frac{\beta_{fm}\;\cdot \beta_{co}}{\beta_{fm}+\beta_{co}\;}$$

where $\beta_{fm}$ is the collision frequency in the free molecular regime [9]:

(5) $$\beta_{fm}=4\sqrt{\frac{\pi k_{B}T}{m_{Ag}}}d_{c}^{2}$$

where $\beta_{co}$ is the collision frequency in the continuum regime [9]:

(6) $$\beta_{co}=\frac{8k_{B}T}{3\mu }\left(1+\frac{2\lambda_{g}}{d_{m}}\left(1.257+0.4\cdot exp\left(-0.78\cdot d_{m}/\lambda_{g}\right)\right)\right)$$

where $k_{B}\;$is the Boltzmann constant, T is the temperature, $d_{c}$ is the agglomerate collision diameter that is equal to $d_{p}$ for spheres and $d_{g}$ for agglomerates (Equations (1) and (2)) to account for their fractal-like structure on β [38], $m_{Ag}$ is the agglomerate mass:

(7) $$m_{Ag}=\rho n_{p}\pi \frac{d_{p}^{3}}{6}$$

where $n_{p}$ is given by:

(8) $$n_{p}=\frac{N_{Ag,0}}{N_{Ag}}$$

where $N_{Ag,0}$ is the initial number density of the spherical primary particles. The collision frequency of polydisperse agglomerates, $\beta_{p}$, is related to $\beta_{m}$ by [21]:

(9) $$\beta_{p}=\left(1.82\pm 0.35\;\right)\cdot \beta_{m}$$

**Figure 2:**
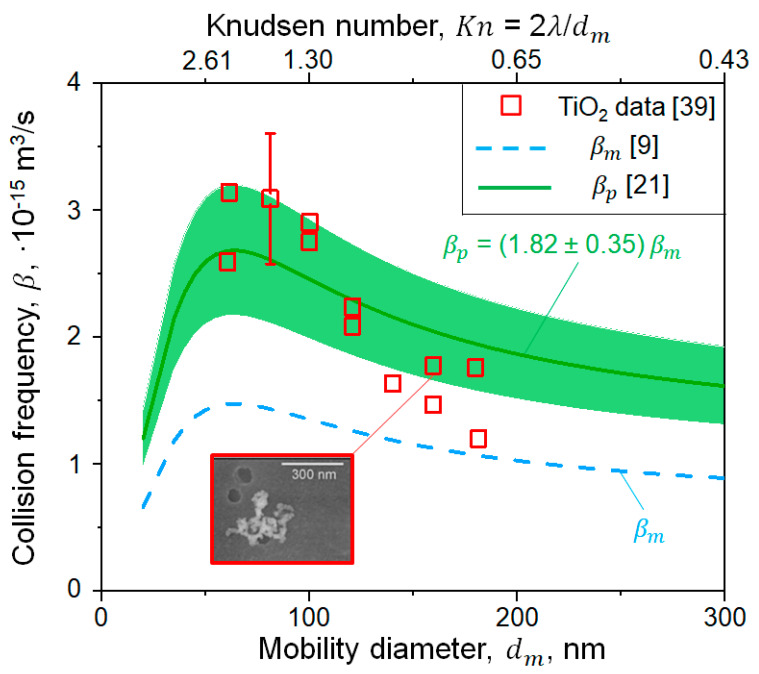
Evolution of collision frequency, β, as a function of agglomerate mobility diameter, $d_{m}$ (bottom abscissa), or Knudsen number, Kn (top abscissa), measured for flame-made TiO_2_ agglomerates (squares, inset) [39] compared to $\beta_{m}$ (Equation (4), broken line) and $\beta_{p}$ (Equation (9), solid line and shade).

The average enhancement factor of *β*_*p*_ and its standard deviation have been derived based on 10 DEM simulations of soot and SiO_2_ nanoparticle agglomeration at temperatures ranging from 1400 to 1830 K and pressures ranging from 1 to 10 bar [21]. Figure 2 shows the evolution of β as a function of $d_{m}$ measured (symbols) or estimated (lines and shade) for monodisperse ($\beta_{m}$, Equation (4); broken line) or polydisperse ($\beta_{p}$, Equation (9); solid line and shade) agglomerates of flame-made uncharged or weakly charged TiO_2_ nanoparticles with *d_p_* = 20 nm at *T* = 295 K [39]. Agglomerates are initially formed by coagulation in the free molecular regime (Kn > 2.6). Therefore, their β rapidly increases with increasing $d_{m}$. Agglomerates with *d_m_* larger than 100 nm coagulate in the transition regime (Kn < 2.6) and their β decreases towards the asymptotic $\beta_{co}$ in the continuum regime (Equation (6)). The measured β is underestimated up to a factor of 2 by $\beta_{m}$ that neglects the polydispersity of the agglomerate size distribution. The Fuchs interpolation for $\beta_{m}$ of monodisperse agglomerates in the transition regime results in a similar underestimation of the measured β [39]. In contrast, $\beta_{p}$ derived for polydisperse agglomerates [21] is 82 ± 35% larger than $\beta_{m}$ and in excellent agreement with the data. This validates the use of Equation (9) in the MPBM of nanoparticle coagulation in the transition regime.

## 3. Results and Discussion

### 3.1. Evolution of Agglomerate Morphology by DEM and a MPBM

**Figure 3:**
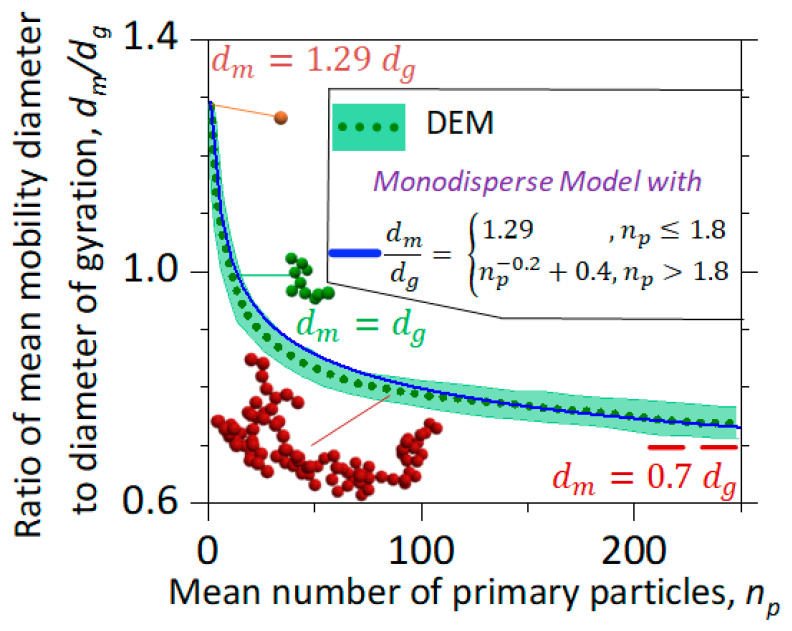
Evolution of the ratio of the mean mobility diameter over that of gyration, $d_{m}/d_{g}$, as a function of $n_{p}$ during agglomeration derived by DEM (dotted line and green shade) and the MPBM interfaced with Equation (2) (solid line). The $d_{m}/d_{g}$ is initially equal to 1.29 for single spheres [40], but rapidly decreases to 1 for agglomerates with $n_{p}\sim 10$ and $\sigma_{g,p}=1$ and asymptotically reaches 0.7 (dashed line) for large agglomerates with $n_{p}\geq 700$ [41]. The DEM-derived relation [10] (solid line, Equation (2)) quantifies the evolution of $d_{m}/d_{g}$ within 6% of the DEM simulations.

Figure 3 shows the dynamics of the agglomerate structure quantified by the ratio of the mean mobility diameter over that of gyration, $d_{m}/d_{g}$, as a function of the mean number of primary particles per agglomerate, $n_{p}$, obtained by DEM (dotted line and shade) and MPBM (solid line). Particles rapidly evolve from spheres with $d_{m}=1.29\;d_{g}$ [40] into agglomerates with $d_{m}=d_{g}$ having $n_{p}\;\sim \;10$, and asymptotically reach $d_{m}=0.7\;d_{g}$ [41] as $n_{p}$ increases. This rapid reduction of $d_{m}/d_{g}$ is induced by the enhancement of the agglomerate inertia that determines $d_{g}$ [42]. Therefore, the agglomerate inertia becomes larger than its drag force at $n_{p}>$ 10 for monodisperse primary particles, resulting in $d_{m}/d_{g}<$ 1. The DEM-derived evolution of $d_{m}/d_{g}$ has been validated with data from wood combustion [10,43]. Most importantly, the agglomerate $d_{m}/d_{g}$ estimated here by the MPBM using Equation (2) is in excellent agreement with that obtained by DEM from first principles. This confirms that the MPBM interfaced with DEM-derived power laws accounts for the agglomerate morphology dynamics in detail. Assuming a fixed value for $d_{m}/d_{g}\;$ in the MPBM, such as 1.29 [18], 1 [15] or 0.7 [41], could significantly reduce its accuracy, as $d_{m}/d_{g}=1.29$ is only valid for spheres and $d_{m}/d_{g}=0.7$ is only reached for very large agglomerates [12]. In this regard, using $d_{m}/d_{g}=1$ in the MPBM results in a 49% overestimation of the agglomerate $d_{m}$, as discussed in Section 3.4.

### 3.2. Impact of Primary Particle Polydispersity on Agglomeration Dynamics

**Figure 4:**
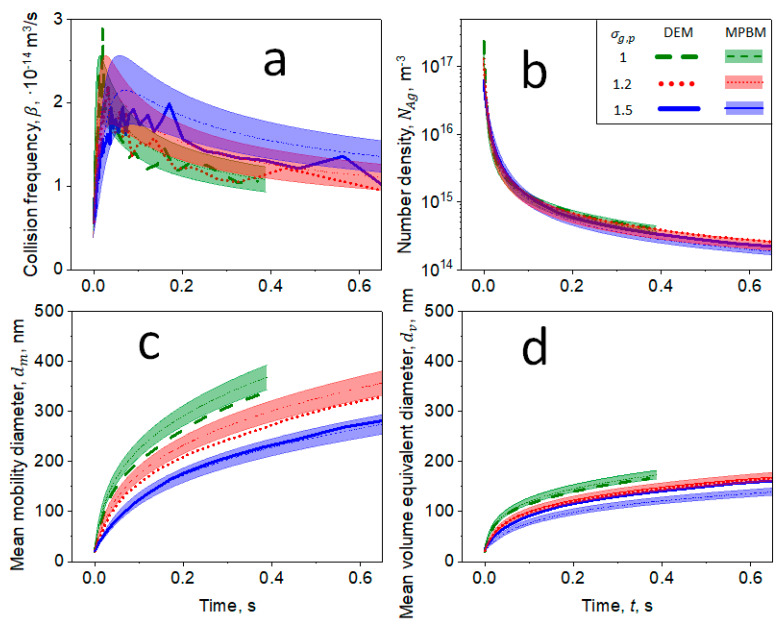
Evolution of (**a**) β, (**b**) number density, $N_{Ag}$, (**c**) mean $d_{m}$ and (**d**) volume-equivalent diameter, $d_{v}$, as a function of time, t, for agglomerates consisting of primary particles with mean $d_{p}=20\;\mathrm{nm}$ and geometric standard deviation, $\sigma_{g,p}$ = 1 (broken lines), 1.2 (dotted lines) and 1.5 (solid lines) at *T* = 1830 K and P=1 bar estimated by DEM (thick lines) and MPBM (thin lines and shades). All sub-figures (**a**–**d**) share the same legend.

Figure 4 shows the evolution of (a) collision frequency, β, (b) number density, $N_{Ag}$, (c) mean mobility, $d_{m}$, and (d) volume-equivalent, $d_{v}$, diameters as a function of time, t, for agglomerates consisting of primary particles with mean $d_{p}=20\;\mathrm{nm}$ and geometric standard deviation, $\sigma_{g,p}$ = 1 (broken lines), 1.2 (dotted lines) and 1.5 (solid lines) at *T* = 1830 K and P=1 bar derived by DEM (thick lines) and the MPBM (thin lines and shades). The shades quantify the statistical variation of $\beta_{p}$ (Equation (9)).

Increasing $\sigma_{g,p}$ enhances β, resulting in a faster reduction of $N_{Ag}$ with time as shown in Figure 4a. However, increasing $\sigma_{g,p}$ also delays the attainment of the tranisition regime, as agglomerates consisting of primary particles with larger $\sigma_{g,p}$ have smaller $d_{m}$ and $d_{v}$. This is due to the small primary particles in those agglomerates that have marginal impact on increasing $d_{m}$ and $d_{v}$. The MPBM-derived $N_{Ag}$, β, $d_{m}$ and $d_{v}$ are in excellent agreement with those obtained by DEM for all $\sigma_{g,p}$ investigated here. Therefore, the present MPBM could be used to simulate the coagulation dynamics of organic nanoparticles with rather monodisperse primary particle size distributions [3,35], and also those of metals [44] and metal oxides [22,37] that attain the self-preserving size distribution by coalescence in practical applications. The MPBM-derived agglomeration dynamics are also in excellent agreement with those obtained by DEM for nanoparticles with *d_p_* = 10, 20 and 40 nm (Supplementary Materials: Figure S1). The present MPBM has been also validated with DEM simulations and measurements of non-spherical aggregated soot nanoparticles coagulating in the free molecular regime [32].

### 3.3. Coagulation of Nanoparticles at High Pressures

**Figure 5:**
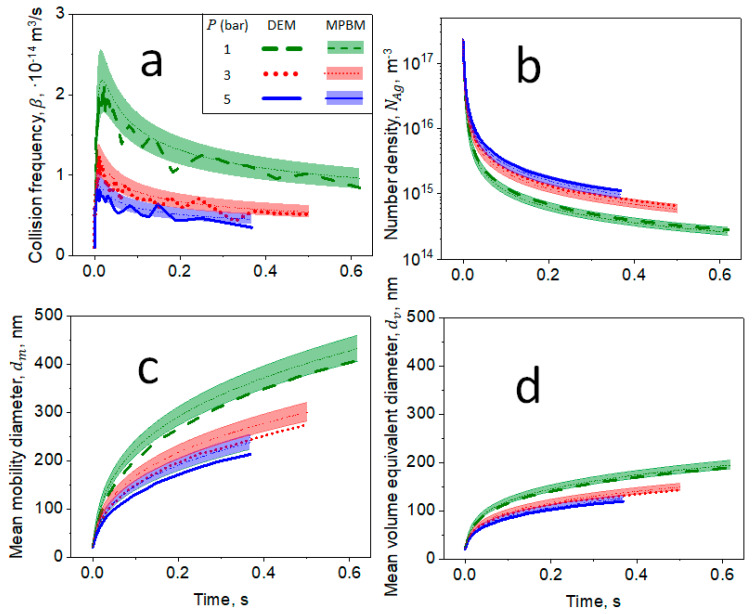
Evolution of (**a**) β, (**b**) $N_{Ag}$, (**c**) $d_{m}$ and (**d**) $d_{v}$ as a function of t for agglomerates consisting of primary particles with mean $d_{p}=20\;\mathrm{nm}$ and $\sigma_{g,p}=1$ at *T* = 1830 K and P=1 (broken line), 3 (dotted line) and 5 bar (solid line) simulated by DEM (thick lines) and MPBM (thin lines and shades). All sub-figures (**a**–**d**) share the same legend.

Figure 5 shows the evolution of (a) β, (b) $N_{Ag}$, (c) $d_{m}$ and (d) $d_{v}$ as a function of t at P = 1 (broken lines), 3 (dotted lines) and 5 bars (solid lines) during the coagulation of monodisperse primary particles with $d_{p}=20\;\mathrm{nm}$ derived by DEM (thick lines) and the MPBM (thin lines and shades). Agglomerates at 1 bar are intially in the free molecular regime. Thus, their β increases as their $n_{p}$ and $d_{g}$ increase rapidly by coagulation. As the agglomerates grow, they enter the transition regime where their β gradually decreases towards an asymptotic value that is rather independent of agglomerate size in the continuum regime [45]. Increasing pressure from 1 to 5 bar decreases β by up to a factor of 2.6, as agglomerate diffusivity decreases at higher pressures [46]. This results in larger $N_{Ag}$ at 3 and 5 bars compared to those obtained at 1 bar. Agglomerate $d_{m}$ and $d_{v}$ derived at 1 bar are at least 50% larger than those obtained at 3 and 5 bar. This is due to their large collistion frequency at 1 bar (Figure 5a) that takes place initially in the free molecular regime. Agglomerate $d_{v}$ is on average 35% smaller than its $d_{m}$ at all pressures, quantifying the ramified non-spherical agglomerate structure [42]. The MPBM-derived $N_{Ag}$, β, $d_{m}$ and $d_{v}$ are in excellent agreement with those obtained by DEM for all P relevant for soot formation in engines investigated here [6]. The MPBM-derived agglomeration dynamics are also in excellent agreement with those obtained by DEM for initial *N_Ag,_*_0_ and (incubation) residence times spanning 6 and 8 orders of magnitude, respectively (Figure S2).

### 3.4. Validation of Low-Temperature Coagulation Dynamics with Experiments

**Figure 6:**
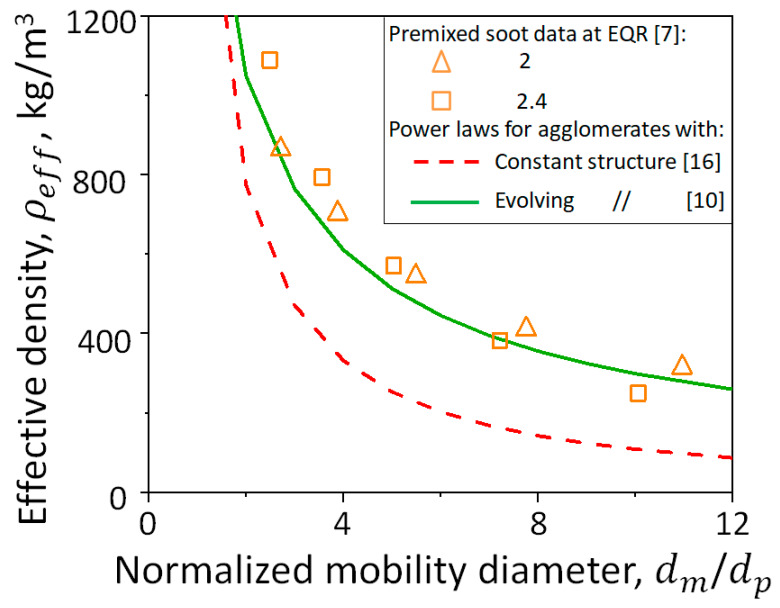
Evolution of agglomerate effective density, $\rho_{eff}$, as a function of their normalized mobility diameter, $d_{m}/d_{p}$, measured for soot particles sampled from premixed ethylene flames with equivalence ratios of 2 (triangles) or 2.4 (squares) and estimated assuming constant (broken line) or evolving agglomerate structure (solid line).

Figure 6 illustrates the agglomerate effective density, $\rho_{eff}$, as a function of the normalized mobility diameter, $d_{m}/d_{p}$, measured for soot particles sampled from premixed ethylene flames with an equivalence ratio (EQR) of 2 (triangles) or 2.4 (squares), and estimated here accounting for the evolving fractal-like agglomerate structure (Equations (1) and (2) [10], solid line) or assuming a constant agglomerate structure with $d_{m}=d_{g}=d_{p}\;n_{p}^{0.56}\;$(broken line) [16]. Ramified agglomerates are formed at EQR = 2 and 2.4 having average $d_{p}=9\;$[35] and 19.6 nm [47], respectively. The agglomerate $d_{m}/d_{p}$ increases during coagulation, reducing $\rho_{eff}$ and $d_{m}/d_{g}$ from 1.29 to 0.7 (as shown in Figure 3). Neglecting the evolving agglomerate structure and assuming $d_{m}=d_{g}=d_{p}\;n_{p}^{0.56}\;$underpredicts the measured $\rho_{eff}$ by up to 50%. In contrast, the agglomerate $\rho_{eff}$ obtained here using DEM-derived power laws (Equations (1) and (2)) that account for the realistic agglomerate morphology is in excellent agreement with data of soot nanoparticles from different combustion conditions. 

**Figure 7:**
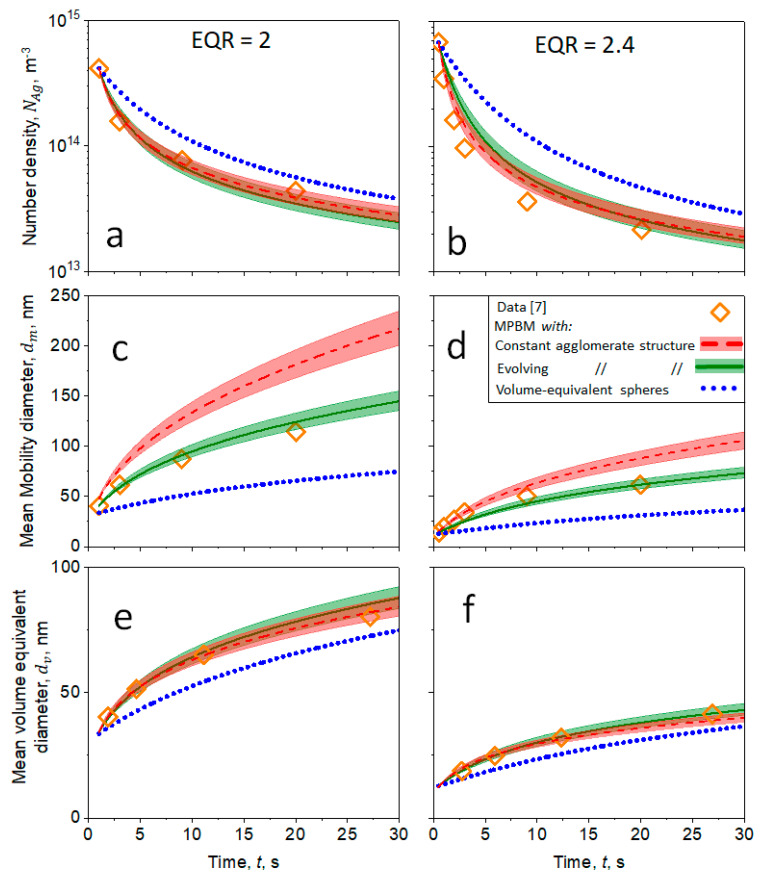
Evolution of agglomerate (**a**,**b**) $N_{Ag}$, (**c**,**d**) $d_{m}$ and (**e**,**f**) $d_{v}$ as a function of *t* measured (diamonds) for soot sampled from premixed flames with EQR = 2 (**a**,**c**,**e**) and 2.4 (**b**,**d**,**f**) [7] compared to those derived by the MPBM (lines, shades) for volume-equivalent spheres (dotted lines) or agglomerates with constant (broken lines) and evolving morphology (solid line). All sub-figures (**a**–**f**) share the same legend.

The impact of the agglomerate morphology on the coagulation dynamics of nanoparticles in the transition regime is investigated next. In particular, Figure 7 shows the agglomerate $N_{Ag}$ (a, b), mean $d_{m}$ (c, d) and $d_{v}$ (e, f) of soot nanoparticles measured as a function of t during coagulation at *T* = 295 K (symbols). Soot nanoparticles are sampled from the premixed flames with EQR = 2 (a, c, e) or 2.4 (b, d, f) shown in Figure 7. The measured $N_{Ag}$, $d_{m}$ and $d_{v}$ (symbols [7]) are compared to those estimated by the MPBM (lines, shades) assuming volume-equivalent spheres (dotted lines) or agglomerates with constant (broken lines) or evolving morphology (solid lines).

Approximating agglomerates with volume-equivalent spheres underpredicts their $d_{m}$ and $d_{v}$ by up to 93 and 18%, respectively, and overpredicts their $N_{Ag}$ by up to 55%. The $N_{Ag}$ and $d_{v}$ obtained by the MPBM for agglomerates with a constant or evolving structure are in excellent agreement with the data of soot obtained from both EQRs. However, neglecting the evolving agglomerate structure during coagulation in the transition regime overpredicts $d_{m}$ by up to 49%. Thus, interfacing the MPBM with DEM-derived power laws (Equations (1) and (2)) is essential to account for the realistic agglomerate structure and accurately estimate the $d_{m}$ dynamics so that they are in excellent agreement with data.

## 4. Conclusions 

A simple monodisperse population balance model (MPBM) is derived here for the agglomeration of nanoparticles in the transition regime. The MPBM uses relations derived from detailed discrete element modeling (DEM) simulations in order to obtain the evolving structure of agglomerates quantified by their mobility and gyration diameters. As a result, the DEM-derived collision frequency, β, that accounts for the agglomerate size polydispersity is 82 ± 35% larger than that of monodisperse agglomerates and in excellent agreement with measurements of flame-made TiO2 nanoparticles. Therefore, the $N_{Ag}$, $d_{m}$ and $d_{v}$ derived by the MPBM, accounting for the evolving fractal-like structure of agglomerates (Equations (1) and (2)) and the impact of their polydispersity on β (Equation (9)), are on par with those obtained by detailed DEM simulations for both monodisperse and polydisperse primary particles coagulating at pressures P = 1–5 bar. Most importantly, the soot agglomerate $N_{Ag}$, $d_{m}$ and $d_{v}$ derived during coagulation at low temperatures are in excellent agreement with data from various premixed flame conditions. In contrast, neglecting the fractal-like morphology of agglomerates or their evolving structure during coagulation results in an underestimation or overestimation of the mean $d_{m}$ by up to 93% or 49%, repectively. Thus, the MPBM derived here accounting for the realistic nanoparticle agglomerate structure can be readily interfaced with CFD in order to accurately simulate the agglomeration dynamics of nanoparticles at high pressures or low temperatures that are present in engines or during sampling and atmospheric aging.

## Data Availability

All the data is available within the manuscript.

## Supplementary Materials

The following are available online at https://www.mdpi.com/article/10.3390/ma14143882/s1, Figure S1: Evolution of (**a**) collision frequency, β, (**b**) number density, $N_{Ag}$, (**c**) mean mobility, $d_{m}$, and (**d**) volume-equivalent, $d_{v}$, diameters as a function of time, t, for agglomerates consisting of monodisperse primary particles with $d_{p}$ = 10 (solid lines), 20 (broken lines) and 40 nm (dotted lines) derived by DEM (thick lines) and MPBM (thin lines and shaded areas) at T = 1830 K and P = 1 bar. The MPBM-derived agglomerate dynamics are in excellent agreement with those obtained by DEM for the wide range of $d_{p}$ studied here. Figure S2: Evolution of (**a**) β, (**b**) $N_{Ag}$, (**c**) $d_{m}$, and (**d**) $d_{v}$ as a function of t for monodisperse primary particles with initial $N_{Ag,0}$ = 2 · 10^15^ (solid lines), 2 · 10^17^ (broken lines) or 2 · 10^19^ m^−3^ (dotted lines) and *dp* = 20 nm derived by DEM (thick lines) and MPBM (thin lines and shaded areas) at T = 1830 K and P = 1 bar. The MPBM-derived agglomeration dynamics are in excellent agreement with those obtained by DEM for $N_{Ag,0}$ and (incubation) residence times spanning 6 and 8 orders of magnitude, respectively.

## Nomenclature

$d_{c}$	Collision diameter, m
$d_{g}$	Gyration diameter, m
$d_{m}$	Mobility diameter, m
$d_{p}$	Primary particle diameter, m
$D_{f}$	Fractal dimension
$D_{fm}$	Mass-mobility exponent
$k_{B}$	Boltzmann constant, $m^{2}\mathrm{kg}/s^{2}/K$
$k_{n}$	Fractal prefactor
$k_{m}$	Mass-mobility prefactor
Kn	Knudsen number
$m_{Ag}$	Single agglomerate mass, kg
$n_{p}$	Number of primary particles per agglomerate
$N_{Ag}$	Agglomerate number density, $m^{-3}$
P	Gas pressure, $\mathrm{kg}/s^{2}/m$
R	Universal gas constant, $m^{2}\mathrm{kg}/s^{2}/\mathrm{mol}/K$
t	Time, s
T	Temperature, K
**Greek letters**	
β	Collision frequency, $m^{3}/s$
ρ	Bulk aerosol density, $\mathrm{kg}/m^{3}$
$\rho_{eff}$	Effective aerosol density, $\mathrm{kg}/m^{3}$
$\sigma_{g,m}$	Geometric standard deviation of $d_{m}$ distribution
$\sigma_{g,p}$	Geometric standard deviation of $d_{p}$ distribution
