# Supplementary material for: A Monodisperse Population Balance Model for Nanoparticle Agglomeration in the Transition Regime
Source: Materials (Basel). 2021 Jul 12;14(14):3882. doi: 10.3390/ma14143882 (PMC8306586; doi:10.3390/ma14143882)
Supplement: Supplementary file 1 [file materials-14-03882-s001.zip › materials-1251691_SM_revised.pdf]

materials

Supplementary Materials: A Monodisperse Population Balance Model for Nanoparticle Agglomeration in the Transition Regime

Georgios A. Kelesidis [1] and M. Reza Kholghy [2,*]

[1] Department of Mechanical and Process Engineering, Eidgenössische Technische Hochschule Zürich, Sonneggstrasse 3, 8092 Zürich,
Switzerland; gkelesidis@ptl.mavt.ethz.ch
[2] Department of Mechanical and Aerospace Engineering, Carleton University, 1125 Colonel by Drive, Ottawa, ON K1S 5B6, Canada
* Correspondence: reza.kholghy@carleton.ca

Citation: : Kelesidis, G.A.; Kholghy, M.R. A Monodisperse Population Balance Model for Nanoparticle Agglomeration in the Transition Regime. *Materials* **2021**, *14*, 3882. https://doi.org/10.3390/ma14143882

Academic Edito: Silvana De Iuliis

Received: 23 May 2021
Accepted: 5 July 2021
Published: 12 July 2021

Publisher's Note: MDPI stays neutral with regard to jurisdictional claims in published maps and institutional affiliations.

Copyright: © 2021 by the authors. Licensee MDPI, Basel, Switzerland. This article is an open access article distributed under the terms and conditions of the Creative Commons Attribution (CC BY) license (http://creativecommons.org/licenses/by/4.0/).

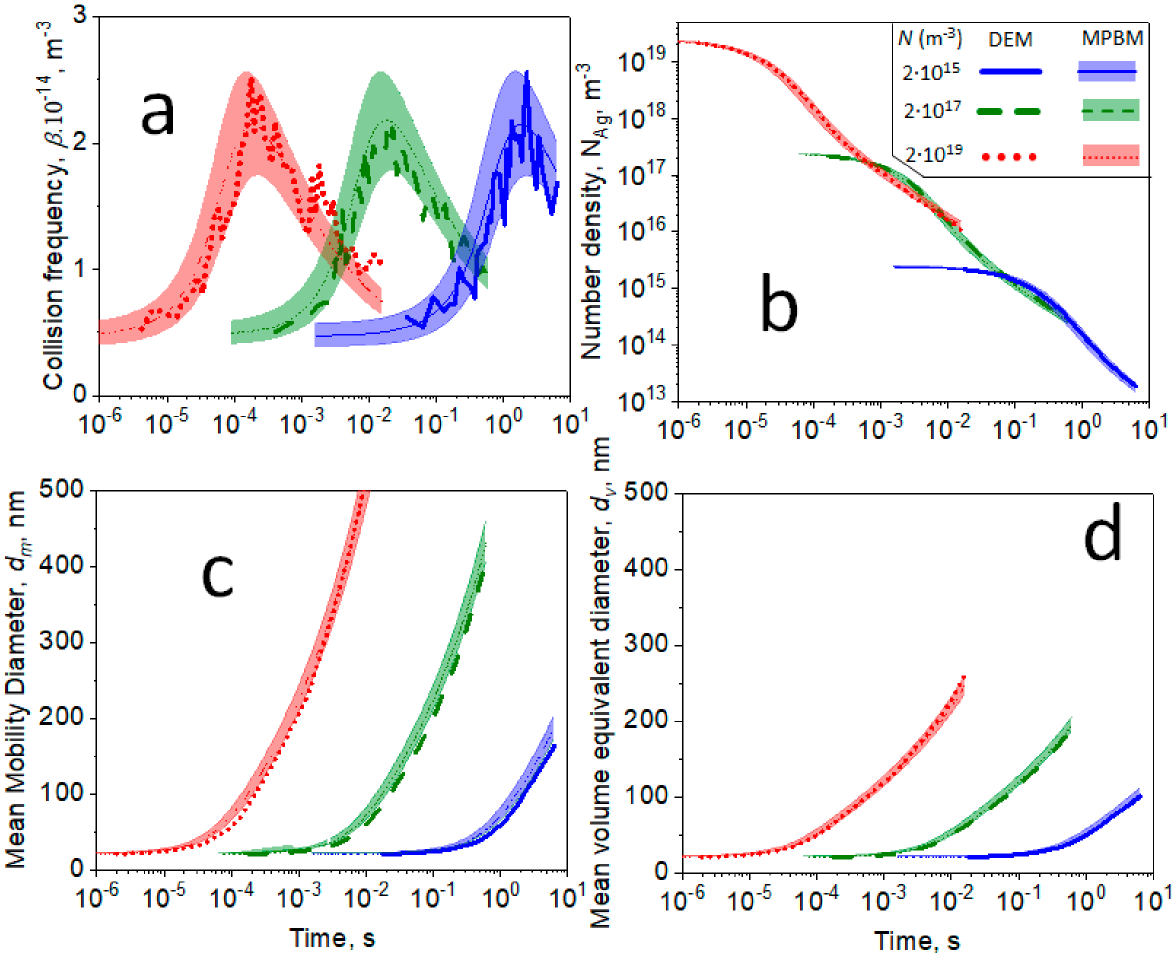

Figure S1. Evolution of (**a**) collision frequency, β, (**b**) number density, N_{Ag}, (**c**) mean mobility, d_m, and (**d**) volume-equivalent, d_v, diameters as a function of time, t, for agglomerates consisting of monodisperse primary particles with $d_p = 10$ (solid lines), 20 (broken lines) and 40 nm (dotted lines) derived by DEM (thick lines) and MPBM (thin lines and shaded areas) at T = 1830 K and P = 1 bar. The MPBM-derived agglomerate dynamics are in excellent agreement with those obtained by DEM for the wide range of d_p studied here.

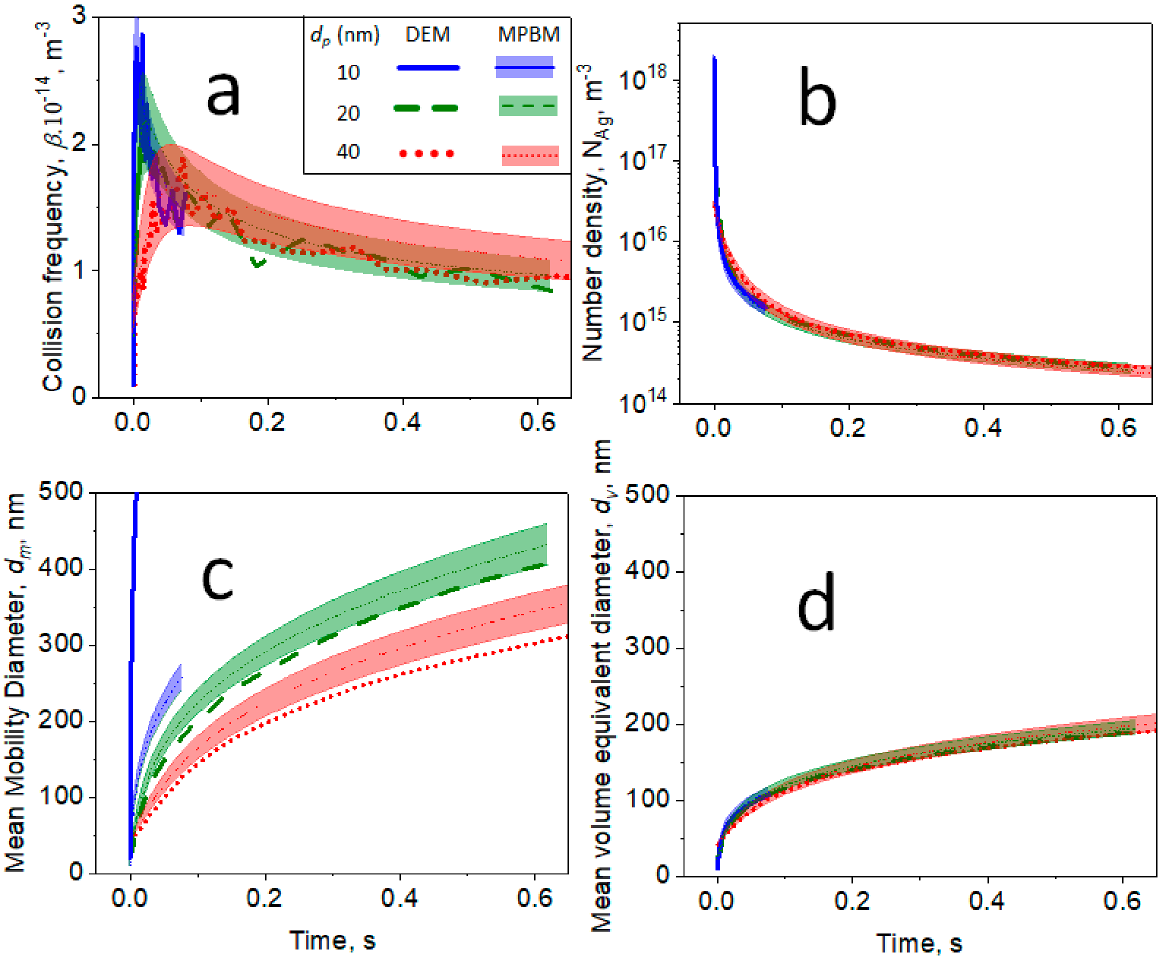

Figure S2. Evolution of (**a**) β, (**b**) N_{Ag}, (**c**) d_m, and (**d**) d_v as a function of t for monodisperse primary particles with initial $N_{Ag,0} = 2 \cdot 10^{15}$ (solid lines), $2 \cdot 10^{17}$ (broken lines) or $2 \cdot 10^{19}$ m^{-3} (dotted lines) and $d_p = 20$ nm derived by DEM (thick lines) and MPBM (thin lines and shaded areas) at T = 1830 K and P = 1 bar. The MPBM-derived agglomeration dynamics are in excellent agreement with those obtained by DEM for $N_{Ag,0}$ and (incubation) residence times spanning 6 and 8 orders of magnitude, respectively.
